# Role of multidrug transporters in neurotherapeutics

**DOI:** 10.4103/0972-2327.53076

**Published:** 2009

**Authors:** Manna Jose, Sanjeev V. Thomas

**Affiliations:** Department of Neurology, Sree Chitra Tirunal Institute for Medical Sciences and Technology, Trivandrum, India

**Keywords:** Drug resistant epilepsy, multidrug resistance, multidrug resistance protein, P-glycoprotein, teratogenic effect, transport proteins

## Abstract

Acquired resistance to antibiotics and other chemotherapeutic agents is a major problem in the practice of neurology and other branches of medicine. There are several mechanisms by which drug resistance is acquired. Multidrug transporters are important glycoproteins located in the cell membrane that actively transport small lipophilic molecules from one side of the cell membrane to the other, most often from the inside to the outside of a cell. They have important protective role yet may prove inconvenient in chemotherapy. In epilepsy and other disorders this mechanism augments the elimination of drugs from their target cells and leads to drug resistance. In this review, we have discussed the biochemical characteristics of multidrug transporters and the mechanisms by which these membrane bound proteins transport their target molecules from one side to the other side of the cell membrane. We have also briefly discussed the application of this knowledge in the understanding of drug resistance in various clinical situations with particular reference to neurological disorders. These proteins located in the placenta have important role in preventing the transplacental movement of drugs in to the fetus which may result in congenital malformations or other defects. The molecular genetic mechanisms that govern the expression of these important proteins are discussed briefly. The potential scope to develop targeted chemotherapeutic agents is also discussed.

It is intriguing how living organisms adapt themselves and evolve strategies to surmount threat from environment. Most of the organisms have their own mechanisms to neutralize the effect of threat from environment. Bacteria and higher organisms can selectively augment their mechanisms to eliminate harmful agents and chemicals from their tissues. Occasionally these mechanisms may interfere with pharmaco therapy of infections, malignancies and various neurological disorders. The cellular mechanisms that preferentially allow or restrict the entry of molecules in to the cells constitute one of the key mechanisms of this surveillance system. ATP-binding cassette (ABC) transporters represent the largest family of transmembrane (TM) proteins that are involved in the transport of molecules across membranes. They have representatives in all extant phyla from prokaryotes to humans, and are classified into seven subfamilies, ranging from ABCA to ABCG. This classification is based on genomic organization, order of domains and sequence homology. P-glycoprotein (PGP) is the best known and most widely studied representative of the ABCB subfamily. PGP confers the ability of multidrug resistance (MDR) phenotype to the organism. It was first discovered in Chinese hamster ovary cells which display cross-resistance to a wide range of amphiphilic drugs. Surface labeling studies performed in these cells showed that the cell membranes of drug-resistant cells possessed a carbohydrate-containing component of 170kDa molecular weight which is not observed in wild type (drug-sensitive) cells. Through a series of studies involving metabolic incorporation of carbohydrate and protein precursors, and selective proteolysis, this component was found to be a cell surface glycoprotein. Juliano and Ling in 1976 designated this as P glycoprotein, which appears unique to mutant cells that displayed altered drug permeability.[1]

P-glycoprotein, encoded by the human MDR1gene, is the first human ABC transporter that was cloned and characterized. In humans, P-glycoprotein was discovered in human KB carcinoma cells that were resistant to various chemotherapeutic agents. There are two other ABC transporters that confer multi-drug resistance to tumors: the multi-drug resistance protein 1 (MRP1, ABCC1), and the mitoxantrone resistance protein (MXR/BCRP, ABCG2). Multidrug resistance protein 1 (MRP1/ABCC1) is an ABC polytopic membrane transporter of considerable clinical importance. It confers multidrug resistance to tumor cells by reducing accumulation by active efflux of the drug. MRP1 is also an efficient transporter of conjugated organic anions. The multidrug-resistance phenotype expressed in mammalian cell lines is complex. Cells quite often acquire cross-resistance to a remarkably wide range of compounds that have no obvious structural or functional similarities. The basis for cross-resistance appears to be decreased net cellular accumulation of the respective drug, and has been attributed to alterations in the plasma membrane.[2]

## Different P-glycoprotein isoforms

Several isoforms of P-glycoprotein have been found in numerous species, including insects, fish, amphibians, reptiles, birds, and mammals. Three isoforms of this protein have been identified in rodents: mdr1a, mdr1b, and mdr2 and two in humans: Class I-MDR1 and Class II- MDR2.[3] The MDR1gene products confer multidrug resistance, whereas MDR2 and mdr2 have physiological functions. It secretes phosphatidylcholine into bile at the bile canalicular membrane of hepatocytes. These isoforms exhibit considerable structural overlap and human and rat gene products show homology of approximately 80%.

## Cellular localization of PGP/MDR1 and MRP1

The role of MDR proteins in the protection against toxic agents is also expressed by their strategic distribution to specific tissues [Table 1]. They are expressed in important pharmacological barriers, such as the brush border membrane of intestinal cells, the biliary canalicular membrane of hepatocytes, and the luminal membrane in proximal tubules of the kidney. MDR proteins are also present in the endothelial cell of the brain capillaries and in the epithelial cells in the choroid plexus, both contributing to the blood-brain barrier (BBB).

**Table 1:** Localization of MRP family members' in different tissues

**MRP member**	**Kidney**	**Liver**	**Testes**	**Brain**	**Intestine**	**Breast**	**Lung**	**Prostate**	**Colon**	**Spleen**
MRP1[8,9]	✓		✓				✓			
MRP2[10–13]	✓	✓			✓					
MRP3[14,15]	✓	✓			✓					
MRP4[16,17]	✓							✓		
MRP5[17,18]				✓						
MRP6[19,20]	✓	✓								
MRP7[21]			✓						✓	
MRP8[22,23]			✓	✓		✓				
MRP9[24,25]	✓	✓	✓	✓		✓				✓

MDR1 is expressed at very high levels in the adrenal gland and kidney; at intermediate levels in the lung, liver, lower jejunum, colon, and rectum; and at low levels in many other tissues, located in the luminal spaces. The *MDR1-* gene is also expressed in several human tumors. The expression of PGP is more restricted to tissues involved in absorption and secretion.[4] PGP is expressed at high levels in the brush border membrane of renal proximal tubules, apical membrane of enterocytes in the gut, bile canalicular membrane in hepatocytes and luminal membrane of brain capillary endothelial cells. Different forms of microglia (ramified, spheroid and phagocytic types) were found to express a functional PGP protein.[5] In polarized cells that form part of any barrier tissue, PGP-MDR1 is localized in the apical (luminal) membrane surface (e.g. in the epithelial cells of the intestine and the proximal tubules of kidney). In mice BBB, mdr1a plays the important role of eliminating toxic drugs and chemicals from the brain. In the absence of PGP in the BBB, certain neurotoxic pesticides like ivermectin and drugs like vinblastine accumulate in the brain to very high levels.[6] The expression of MRP4/Mrp4 in the apical and basolateral membranes of the BBB and choroid plexus, respectively, indicates a role for this transporter in limiting organic anion influx from blood and in driving organic anion efflux from the brain to blood.[7] It has also been reported that MXR is highly expressed in the placenta, liver and in various stem cells.[25,26]

## Structure of ABC transporters

**Figure 1:**
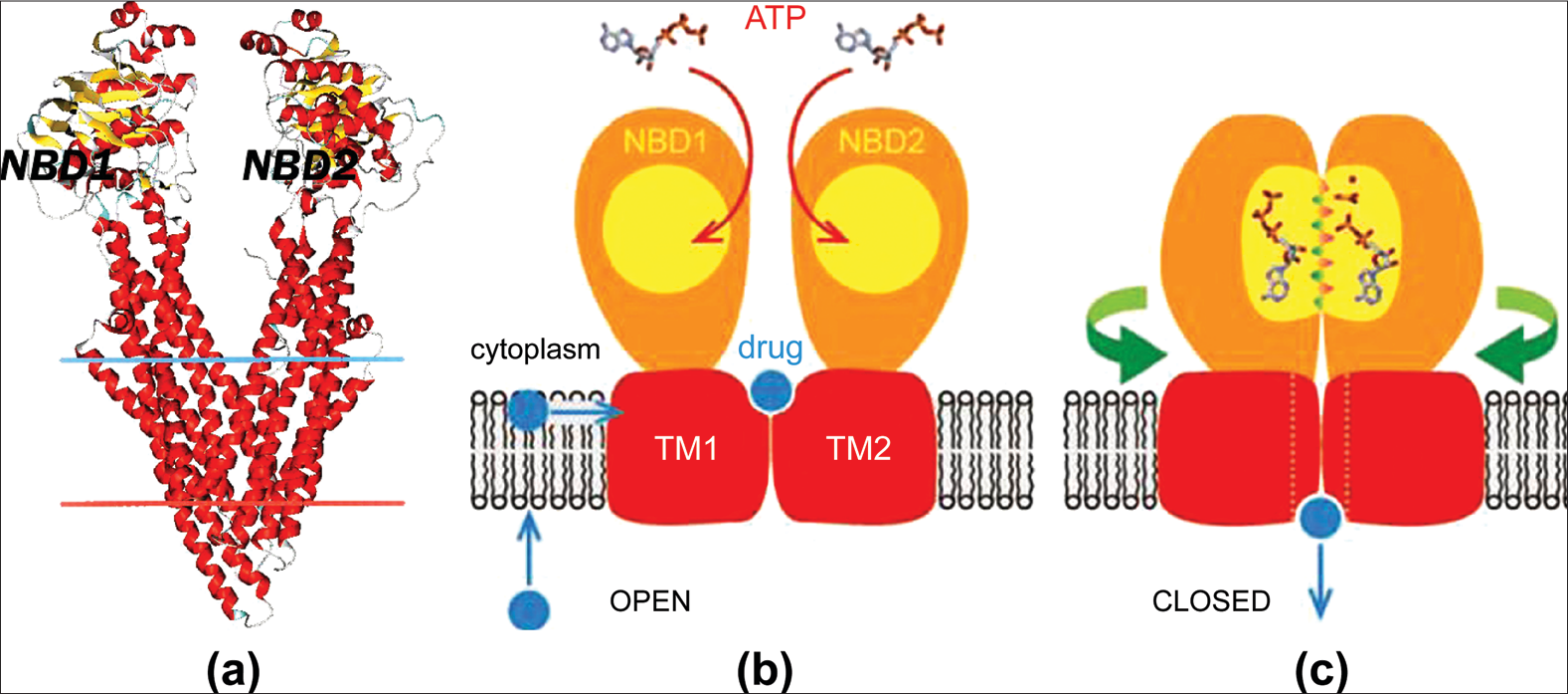
(a) P-glycoprotein structure showing the two transmembrane domains and two nucleotide binding domains. Figure is extracted from the Orientations of Proteins in membranes (OPM) database (ID 3g5u). (b and c) Diagrammatic representation of a likely mechanism of P-glycoprotein action involving dimerization of the nucleotide binding domains. On the left is the Open form of PGP. ATP binds loosely to both NBDs. Drugs have high affinity for PGP and binds from the inner membrane leaflet. When both ATP-binding sites and the drug-binding site are occupied, the PGP acquires Closed form (right). Here both NBDs fix into each other with catalytic side-chains from NBD1 inserting into NBD2 and vice versa. The catalytic transition state forms around one of the two nucleotide _-phosphates, and loss of this transition state is accompanied by Pi release and this leads to loss of affinity of the drug for PGP.[modified from ref 44]

P-glycoprotein consists of 1280 amino acids. It is classified as ABC transporter based on the sequence and organization of the ATP-binding domain(s), also known as nucleotide-binding folds (NBFs). The NBFs contain characteristic (Walker A and B) m otifs, separated by approximately 90-120 amino acids. ABC protein also contain an additional element, the signature (C) motif, located just upstream of the Walker B site. The functional PGP typically contains two NBFs and two transmembrane (TM) domains. The TM domains contain 6-11 membrane-spanning α-helices and provide the specificity for the substrate. The NBFs are located in the cytoplasm and transfer the energy to transport the substrate across the membrane. ABC pumps are mostly unidirectional. In eukaryotes, most ABC proteins move compounds from the cytoplasm to the outside of the cell or into an intracellular compartment [endoplasmic reticulum, mitochondria and peroxisome].[26] Dawson and Locher in 2006 crystallized the outward-facing conformation of the ABC protein in the ATP-bound state, when the 2 nucleotide-binding domains are in close contact and the 2 transmembrane domains form a central cavity [Figure 1]. In this state, the drug translocation pathway is shielded from the inner leaflet of the lipid bilayer and from the cytoplasm, but is exposed to the outer leaflet and the extracellular space.[27]

MRP family contains 13 members, of which nine members can be further divided into two types based on putative membrane topology.[28] MRP1 to MRP3, MRP6 and MRP7 contain three transmembrane domains. In addition to an MDR1-like core, MRP1 contains an additional N-terminal segment of about 280 amino acids. A major part of this region is membrane-embedded with five transmembrane helices, while a small cytoplasmic loop of about 80 amino acids (L0) connects this area to the core region. The presence of the L0 region (together with the core region) is necessary for both the transport activity and the proper intracellular routing of the protein.[29,30] MRP4, MRP5, MRP8 and possibly MRP9 are considered to be “short” MRPs, as they do not contain TMD but do retain the cytoplasmic linker.

ABCG2 (MXR/BCRP) is a half transporter with a unique domain arrangement, where the ABC is located at the N-terminus. This protein, in contrast to many other ABC half-transporters is localized in the plasma membrane. In the N-glycosylated mature form it extrudes positively charged molecules from the cells.[25,31]

### Molecular mechanism of the multi-drug pumps

PGP acts like a hydrophobic vacuum cleaner or flippase [Figure 1]. It intercepts the drug as it moves through the lipid membrane and flips the drug from the inner leaflet of the plasma membrane lipid bi-layer to the outer leaflet and into the extracellular medium.[32] Chemicals transported by PGP have very diverse structures that share only the properties of being hydrophobic amphipathic molecules. They include anti epileptic drugs like phenytoin, phenobarbitone, lamotrigene and levetiracetam anti-cancer drugs such as doxorubicin, vinblastine, immunosuppressive drugs such as cyclosporine A; steroids like hydrocortisone, cortisol, anti-HIV drugs such as ritonavir and saquinavir; cardiac drugs such as digoxin and quinidine; the lipid-lowering agent lovastatin.[33–35]

Drug transport by MDR proteins requires the energy of ATP-hydrolysis, controlled by drug interaction, and closely coupled to the actual drug translocation. Interaction with the drug-substrate significantly enhances the rate of ATP cleavage.[36–38] The site(s) of multi-drug transporters interacting with the drug-substrates are probably encoded in the transmembrane (TM) domains. Detailed mutagenesis studies revealed that TM helices 5 and 6 (in the N-proximal TM domain), helices 11 and 12 (in the C-proximal TM domain), as well as the short cytoplasmic loops connecting these helices, are involved in the formation of an extended drug-binding site(s).[39] It is also strongly indicated that the hydrophobic substrates of MDR1 are recognized within the membrane bilayer or in its vicinity. A similar picture has also emerged in the case of MRP1.[40]

The ATP-hydrolytic cycle of both MDR1 and MRP1 have been investigated in detail. It has been documented that the interaction of the two ABC units is an essential requirement for the catalytic reaction.[41,42] Several lines of evidence indicate that both NBDs can bind ATP and both catalytic sites are active and at least in the case of MDR1, the two ABC domains enter alternately into the catalytic cycle.[43] The hydrolytic step triggers conformational changes, which reduces drug binding to the actual binding site (and presumably makes binding to another site favorable, from which the drug can be released to the extracellular space). Bodo *et al.* demonstrated that hydrolysis of a second ATP-molecule is required for conformational changes to reset the MDR1 transporter to the high affinity drug-binding conformation. On the basis of these results it appears that two ATP molecules are cleaved per molecule of drug substrate transported.[44]

## General Pharmacological Role and Medical Significance of the MDR Proteins

*Biological barrier to xenobiotics*- ABC proteins bind ATP and use that energy to drive out the various molecules across the cell membranes. Most of the known functions involve the shuttling of hydrophobic compounds either within the cell as part of a metabolic process or to outside the cell for transport to other organs or for secretion from the body.[24] The major physiological role of multidrug transporters, especially PGP, is the protection of cells and tissues from xenobiotics.[44] DDT, an organochlorine pesticide and its metabolite, p,p'-DDE's induces *MDR1* gene function as a defence against xenobiotic exposure.[45] Similarly, MDR1/PGP expression is severely increased by plastic derived xenobiotics[46] and can decrease toxicity by removing environmental toxicants such as heavy metals from cells in mammals. Enhanced transporter expression in normal hepatocytes[47] and BBB[48] in response to environmental and chemotherapeutic toxins may have a protective role by limiting xenobiotic exposure to cells.

*Multiple drug resistance*-PGP transports large hydrophobic, uncharged or slightly positively charged compounds. *Anticancer drugs*: PGP transports a wide variety of functionally and structurally diverse cytotoxic drugs out of tumor cells. Thus they are key molecules that cause multidrug resistance in cancer.[49] Overexpression of P-glycoprotein appears to be a consistent feature of mammalian cells displaying resistance to multiple anticancer drugs and has been postulated to mediate resistance.[50] Tumors arising from tissues where MDR1/PGP is highly expressed show intrinsic resistance to chemotherapy.[51] It is also reported that the intercellular transfer of functional PGP from PGP-positive to PGP-negative cells *in vitro* and *in vivo* mediates the acquired multidrug resistance in tumor cells.[52] Shen *et al* showed that in human carcinoma cells, multidrug resistance to colchicine, vinblastine, or adriamycin is associated with amplification of multidrug resistance locus.[53] Increased expression and amplification of MDR1 sequences were found in multidrug-resistant sublines of human leukemia and ovarian carcinoma cells.

*Antiepileptic drugs*: Nearly 30% of patients with epilepsy eventually develop resistance to antiepileptic drugs. This is an unresolved problem and mechanisms of intractability are not completely understood. It has been hypothesized that overexpression of PGP and other efflux transporters in the cerebrovascular endothelium in the region of the epileptic focus may lead to drug resistance in epilepsy. This hypothesis is supported by several findings. In patients with drug resistant epilepsy there is increased expression of efflux transporters within the epileptic foci. In animal models seizures have been found to induce increased expression of PGP. Recently it had been shown that some commonly used AEDs are substrates to the PGP.[54]

MDR has also been shown to play a role in the migration of dendritic cells.[5] MRP family primarily transports hydrophobic anionic conjugates and extrudes hydrophobic uncharged drugs.[55] MRP1 may play an essential role in maintaining glutathione (GSH) concentrations and redox balance of astrocytes during oxidative stress.[56,57] The expression patterns of MRP1 and glutamyl cysteine synthetase, the rate-limiting enzyme of GSH synthesis, may be regulated by oxidative stress and heavy metals.[58–60] Transport of conjugated compounds and the oxidized form of GSH (GSSG), in addition to possible up-regulation of GSH-synthesizing enzymes, strongly suggests a role for MRP1 in detoxification and “phase III” elimination of toxic endogenous metabolites.[61]

MRP2[62] and MRP3[62] probably play important roles in excreting metabolites into the bile. MRP4-transfected cells show increased efflux of monophosphorylated nucleotides and nucleotide analogs such as cAMP, cGMP, and PMEA. Interestingly, GSH stimulates transport of unconjugated bile acids (cholyltaurine, cholylglycine, and choline) from hepatocytes, suggesting that some MRP4 substrates may require GSH for efficient transport.[63] Both MRP4 and MRP5 transport monophosphorylated compounds, such as Phosphonyl Methoxy Ethyl Adenine (PMEA)[64–66]

### Allelic variants and functional polymorphisms of the human multidrug resistance gene

**Figure 2:**
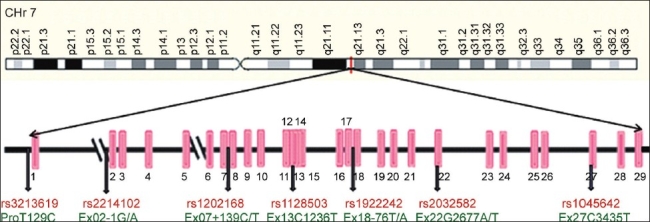
MDR1 gene organization pattern showing the exons (pink bars), including polymorphisms in the gene denoted by the arrows showing their location in the gene, along with their SNP IDs (in red) and name of polymorphism (in green)

MDR-1 gene is mapped to chromosome 7q21.1 and is composed of 28 exons ranging in size from 49 to 209 base pairs and the encoded mRNA is of 4.5 kb size [Figure 2]. In 1987, Ueda and co workers had successfully constructed a full-length cDNA copy of human MDR gene and inserted it into a retroviral expression vector which in turn acquired complete multidrug-resistance phenotype.[67]

The “overall MDR-1 activity” that constitutes the PGP-dependent drug transport depends on the expression of the MDR-1gene which in turn controls the amount of PGP that is synthesized in the cells.[68] Allelic differences in individual MDR1 gene sequences may influence expression levels. There are several important locations in the human MDR1 gene, where a sequence difference could alter its expression levels. The promoter and/or enhancer region, sequences that influence the mode or efficacy of processing of the pre-mRNAs, and sequences that influence mRNA stability are such locations[69–71]

Hoffmeyer and colleagues in 2000 had described 15 polymorphisms in the MDR-1 gene in a human population[68] One of these polymorphisms showed significant correlation with MDR1 expression levels and PGP activity *in vivo*. PGP expression and function in the duodenum was determined by Western blot and quantitative immunohistology and by measuring plasma concentrations after oral administration of digoxin. A 3435C-T transition in exon 26 of MDR1 correlated with expression level and function. Individuals homozygous for this polymorphism had significantly lower duodenal MDR1 expression and the highest digoxin plasma levels. Homozygosity for this variant was observed in 24% of 188 individuals studied. Thus, this polymorphism is expected to affect the absorption and tissue concentrations of numerous other substrates of MDR1.

Different research groups have detected a total of 50 SNPs and insertion/deletion polymorphisms in the cDNA[72,73] A total of 25 single nucleotide polymorphisms (SNPs) in the exonic regions of the *ABC* 1 gene were identified by sequencing, most of them were located in the region between bp 150,000 and 200,000[74] Ten of the 25 exonic polymorphisms detected are synonymous polymorphisms (the resultant codon also codes for the same aminoacid as the wild form) and 15 are non-synonymous. Of these 25 polymorphisms, 16 are newly identified SNPs. Fifty-six of the ninety-five American samples studied had the non-synonymous 2677G>T/A/C polymorphism, resulting in amino acids serine, threonine and proline, respectively. Five individuals had a non-synonymous SNP at site 2959, resulting in an Alanine to Proline alteration (the latter usually encodes a helix termination). All non-synonymous SNPs are intracellular and none are found to appear at ATP binding sites.

Schwab *et al*., in 2003 described that many variants in the *MDR-1* gene are silent (synonymous) polymorphisms and do not produce a change in amino acid sequence. The SNP at location 3435C>T plays a significant role in prediction of haplotypes in the *ABC* 1 gene[75]

There is wide variation in the prevalence of the 3435C/T polymorphism in various ethnic populations: 83% of West Africans and 61% of African Americans were homozygous for the C allele, whereas only 26% of Caucasians and 34% of Japanese showed this genotype[76] The frequency of the C allele was 90% in Ghanaians compared with 50% in Caucasians. People who are homozygous for the 3435T allele of MDR1 were found to have substantially lower intestinal PGP expression than those homozygous for the C allele. HIV patients of African origin are likely to show relative resistance to protease inhibitors and cyclosporine A because of the higher expression of MDR1 pump that eliminates these drugs from their body.

### Role of MDR1 polymorphisms in drug-resistant epilepsy

Although the prognosis for the majority of patients with epilepsy is good, up to 30% of patients continue to have seizures despite carefully optimized antiepileptic drug (AED) treatment[77] Two principal theories, alterations in drug penetration to the brain and alterations in drug targets, have been suggested to play a role in therapeutic failure in this population[78] Several studies have demonstrated a significant association between MDR1 polymorphisms and PGP expression in patients with drug-resistant epilepsy [Table 2].

**Table 2:** Studies demonstrating association between MDR1 polymorphisms and drug-resistant epilepsy

**Study group**	**Sample**	**Polymorphisms screened**	**Results**
Siddiqui *et al.*,[79]	200 patients with drug-resistant epilepsy and 115 patients with drug-responsive epilepsy	3435C/T	3435 C/C genotype frequency increased in patients with drug-resistant epilepsy compared to T/T genotype
Tan *et al.*,[80]	401 drug-resistant and 208 drugresponsive patients with epilepsy	3435C/T	No association between the C/C genotype and drug-resistant epilepsy
Zimprich *et al.*,[81]	210 patients with mesial TLE and 228 controls	1236C/T, 2677G/T, 3435C/T	CGC haplotype in patients with higher pharmacoresistance
Sills *et al.*,[82]	170 patients were responders and 230 non-responders	3435C/T	No association between polymorphism and pharmacoresistant epilepsy
Tate *et al.*,[83]	281 and 425 epileptic patients treated with PHT and CBZ, resp.	3435C/T	No association b/w polymorphism and maximum drug dosage
Kim DW *et al.*,[84]	108 patients with drug-responsive epilepsy, 63 patients with drug-resistant epilepsy and 219 control migraine subjects	3435C/T	No significant association b/w the CC genotype and the multidrug-resistant epilepsy
Kim YO *et al.*,[85]	99 drug resistant and 108 drug responsive persons with epilepsy	1236C/T, 2677G/T, 3435C/T	Genotype and haplotype frequencies in the resistant group were not statistically different than responsive group
Chen *et al.*,[86]	164 drug-responsive and 50 drugnonresponsive children with epilepsy	3435C/T	No association b/w CC genotype or C allele and response to AED treatment
Shahwan *et al.*,[87]	242 drug-responsive and 198 drugresistant persons with epilepsy	Combination of 8 SNPs	No association of drug-resistant epilepsy with C3435T, nor any other functional variants at SNP or haplotype level
Ozgon *et al.*,[88]	97 patients treated with CBZ and 174 healthy individuals	3435C/T	No association b/w polymorphism and resistance to CBZ

Several other groups have also studied the expression levels and cellular distribution of different multidrug resistance proteins in different populations. Increased MDR1 expression had been observed in the epileptogenic zone in the surgically excised specimen from patients with intractable temporal lobe epilepsy. Increased immunohistochemical staining of expression of PGP had also been demonstrated through immunohistochemical staining of this tissue. It appears that seizures may induce selective increase in the expression of MDR genes in strategically important locations in the brain.[89]

MDR1 might have different roles depending on the location in the brain where it is expressed. Over expression of MDR1 in endothelial cells would result in reduced penetration of AEDs into the brain while, MDR1 when overexpressed in the parenchyma would extrude xenobiotics and other drugs from the intracellular compartment and thereby reduce the concentration of the drug within the cells. It is observed in studies where intracellular levels of phenytoin were estimated that MDR1 is highly expressed in vessels of the blood brain barrier and astrocytes and neurons in drug refractory epilepsy. In this study it was shown that phenytoin was significantly lower in astrocytes from epileptic tissue than in control astrocytes.[90] In a recent study, the normal brain showed no expression of MDR1 in the neurons or astrocytes while intense overexpression of MDR1 and MRP1was demonstrated in the neurons and the reactive astrocytes in the dysplastic tissues that were resected from patients with focal cortical dysplasia.[91]

PGP expression in the intestinal mucosa would also influence the absorption and bioavailability of AEDs. The intestinal expression levels and genetics of MDR1 and MRP2 were correlated with dose requirement and plasma levels of carbamazepine (CBZ) and phenytoin (PHT). In this study, 29 patients with epilepsy who were on CBZ and 15 patients on PHT were stratified into a ‘high’-dose (CBZ > or =800mg/day, PHT > or =300mg/day) and a ‘low’-dose group (CBZ < or =600mg/day, PHT < or =200mg/day). Their DNA was obtained by duodenal biopsy for Western blotting and genotyping studies. Low CBZ plasma levels showed a trend towards higher intestinal MDR1 expression.[92]

The expression of MRP tends to be higher in patients with refractory epilepsy as in focal cortical dysplasia and temporal lobe epilepsy. Immunocytochemical studies have positively identified the MRP1 protein in dysplastic neurons, reactive astrocytes and glial elements of focal cortical dysplasia of malformations commonly observed in refractory epilepsy, i.e., human focal cortical dysplasia, dysembryoplastic neuroepithelial tumors and hippocampal sclerosis samples.[93–95] In these studies, MRP1 staining was more prominent in the epileptic lesions, compared with surrounding normal tissue samples. MRP2 and MRP5genes are upregulated in temporal lobe tissues from patients with refractory temporal lobe epilepsy when compared to similar specimen from persons without epilepsy.[96] Dysembryoplastic neuroepithelial tumors from patients undergoing AED treatment with various combinations of carbamazepine, oxcarbazepine, tiagabine, and lamotrigine also exhibited increased MRP2 and MRP5 protein expression, compared with peritumoral tissue or samples obtained from patients diagnosed with arteriovenous malformations.[97]

### Significance of multidrug resistant proteins in teratogenicity

Localisation of multidrug resistant proteins in the human placenta is important for protecting the fetus from unintended, harmful drug exposure, but also for limiting the access of drugs to the fetus after maternal drug intake. P-glycoprotein, MRP family members and BRCP provide mechanisms that protect the developing fetus. MRP1 is localized to the endothelium of the vessels. PGP and MRP2 were detected in the apical membranes of the syncytiotrophoblast and not in fetal blood vessels.[98] (These transporters located in the placenta would pump out the drugs and similar molecules from the fetal side in to the maternal blood and keep the infant protected.

The MDR1 polymorphisms (C3435T and G2677T) are likely to have an impact on P-glycoprotein expression in the human placenta.[99] MDR1 mRNA and P-glycoprotein were analysed in 73 full-term human placentas of Caucasians, as well as respective MDR1genotypes/haplotypes, for the C3435T and G2677T/A polymorphisms of mothers and infants. In this study, the MDR1 mRNA levels were not different between these genotype groups. However, P-glycoprotein expression was significantly lower when both mother and infant were homozygous for the 3435T allele (TT/tt) compared to maternal and fetal homozygotes for the C-allele (*P* = 0.01). Moreover, placentas from mothers carrying both polymorphisms (3435T and 2677T; TT/TT) also had a significantly lower P-glycoprotein expression compared to placentas of wild-type individuals.

The expression of these proteins vary according to the gestational age and the prevailing hormonal milieu. It is observed that the levels of Abcb1a and Abcb1b mRNA were measured and were found to be high at midgestation, and thereafter to decline. A significant correlation between maternal plasma progesterone concentration and placental Abcb1b mRNA, but not Abcb1a mRNA, levels also was demonstrated. Progesterone has been shown to interact with, but not transported by ABCB1.[100]

An increased amount of MRP5 mRNA was detected in second trimester human placentas in comparison with term placentas. Immunofluorescence microscopy with an anti-MRP5 antibody indicated localization of MRP5 preferentially in the basal membrane of syncytiotrophoblasts and in and around fetal vessels. In term placentas some mRNA expression was observed, in addition, in the apical membrane of the syncytiotrophoblast.[101] Tate *et al*., identified polymorphism in the promoter of the *MDR1* gene and investigated their effects on transcriptional activity and mRNA expression in the placenta. Among these variants, the promoter haplotypes containing T-1517aC, T-1017aC, and T-129C were particularly associated with high level of transcriptional activity and mRNA expression but independently of the coding SNP C3435T.[83]

### MDR modifying agents

Prevention of clinical MDR should significantly improve therapeutic response in a large number of patients resistant to a variety of drugs. One way to achieve this goal would be to develop therapeutic agents that do not interact with any of the drug transporters. There are several pathways that prevent the expression or function of multi-drug transporters, but pharmacological modulation seems to be the first choice at present. MDR modifying agents, which inhibit the function of the MDR proteins either competitively or non-competitively, are good candidates for such a pharmacological modulation. These compounds are expected to increase the cytotoxic action of MDR-related drugs by preventing the extrusion of anticancer drugs from the target cells. The co-application of a non-toxic “MDR-modulating” compound with combination traditional drugs may significantly improve the cure rate for cancer and epilepsy. Indeed, we have witnessed a growing interest in the use of such clinically active agents. Biochemical investigations led to the identification of several PGP modulator compounds, as diverse in structure as the transported drugs. Calcium channel blockers like verapamil, diltiazem, azidopine, quinine derivatives, calmodulin inhibitors and the immunosuppressive agent, cyclosporin A, have been shown to interact with the MDR transporters *in vitro* and *in vivo.*[102]

The second generation modulators consisted of derivatives of these first generation compounds, which had less pronounced effect on their primary target, but retained their modulatory effects. Two prominent examples of this group may be R-verapamil and PSC-833, the latter being a cyclosporin analog without immunosuppressive effect. Because of the differences in the substrate-specificities and inhibitor-sensitivities of the different MDR-ABC proteins expressed in different cells, proper therapeutic intervention requires an advanced diagnosis and selection of appropriate targeted modulator agents.

The third generation MDR modifiers are molecules specifically devised to interact with specific MDR transporters as these agents are mostly in the early development phase, their clinical efficacy has yet to be proven.[32,103]

A distinct mode of MDR reversal includes the use of monoclonal antibodies. Several antibodies have been demonstrated to inhibit *in vitro* PGP/MDR1 mediated drug efflux.[104,105] Antibodies that bind to extracellular epitopes of the transporter may provide a reversal of MDR. Another potential method to eliminating MDR is the use of macromolecular carriers. Conjugation of drugs to various drug carriers has a wide range of application (induction of immune response, production of epitope-specific antibodies, etc.) and anti-tumor agents can efficiently bind to such carriers.

In conclusion, PGP is an important component of BBB and placenta barrier, and functions as a protective biological barrier by extruding toxins, drugs, and xenobiotics out of the cell. It not only causes multidrug resistance in cancer but it has also been found to be responsible for MDR in epilepsy and many other diseases. Targeted inhibition of PGP may represent an important strategy by which this serious therapeutic limitation can be overcome. Inhibition or induction of PGP by various drugs or herbs can lead to significant drug-drug or drug-herb interactions, thereby changing the systemic or target tissue exposure of the drug. It is increasingly being recognized to play an important role in process of absorption,[106] metabolism,[107] and excretion[108] of many clinically important drugs in humans. Because of its importance in pharmacokinetics, PGP transport screening can be incorporated into the drug discovery process.

It is clear that drug interaction mediated by PGP may have a great impact on drug disposition particularly regarding brain and placenta. Genetic polymorphism of PGP has also been recorded recently, which may affect drug disposition and produce variable drug effects. Modifying the PGP expression can be one potential mechanism by which resistance to drugs can be overcome in the management of epilepsy, malignancy and several other disorders.
